# Supplementary figures and images for: Seasonal and diel variations in the vertical distribution, composition, abundance and biomass of zooplankton in a deep Chilean Patagonian Fjord
Source: PeerJ. 2022 Jan 25;10:e12823. doi: 10.7717/peerj.12823 (PMC8796712; doi:10.7717/peerj.12823)

A

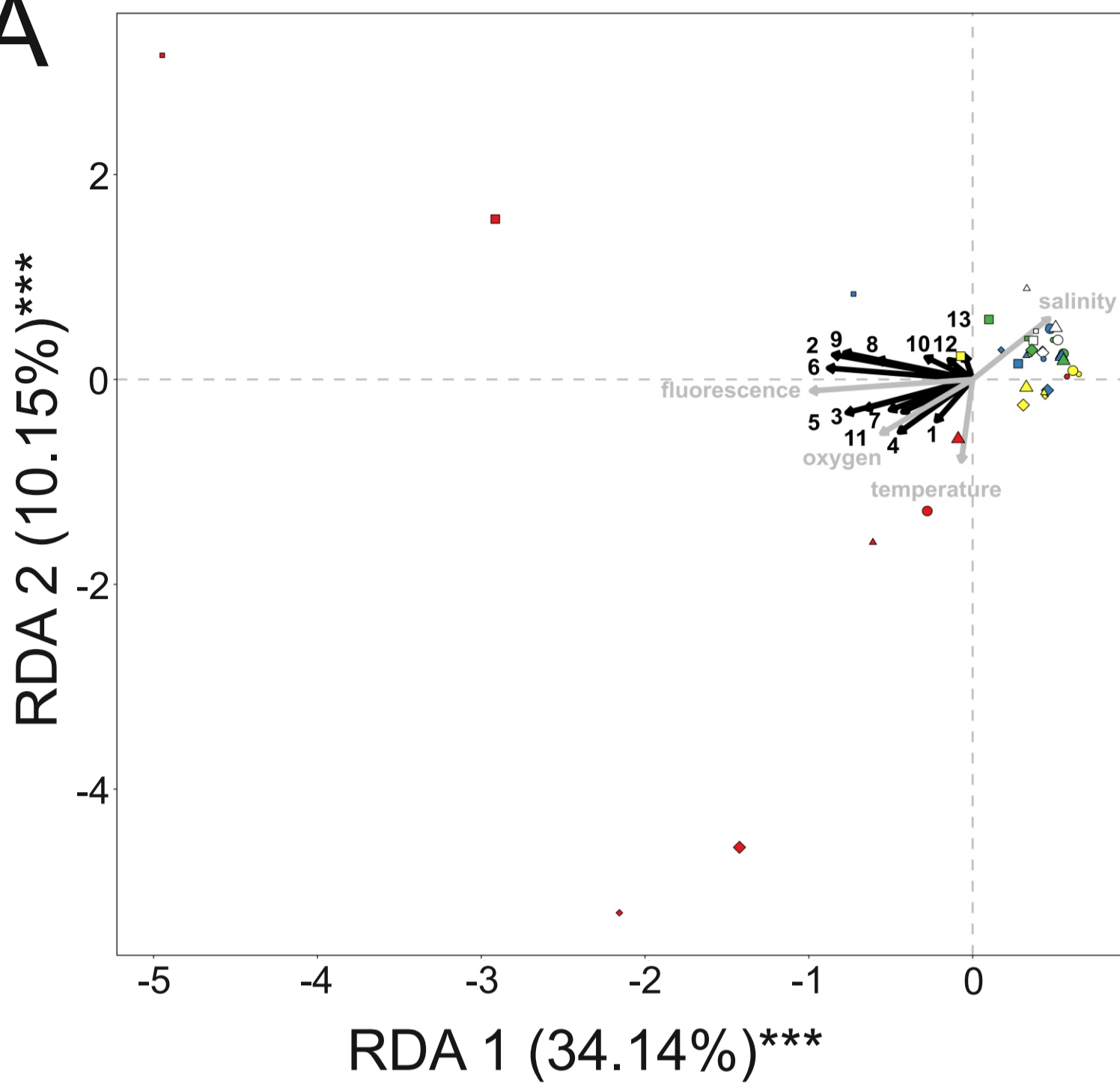

B

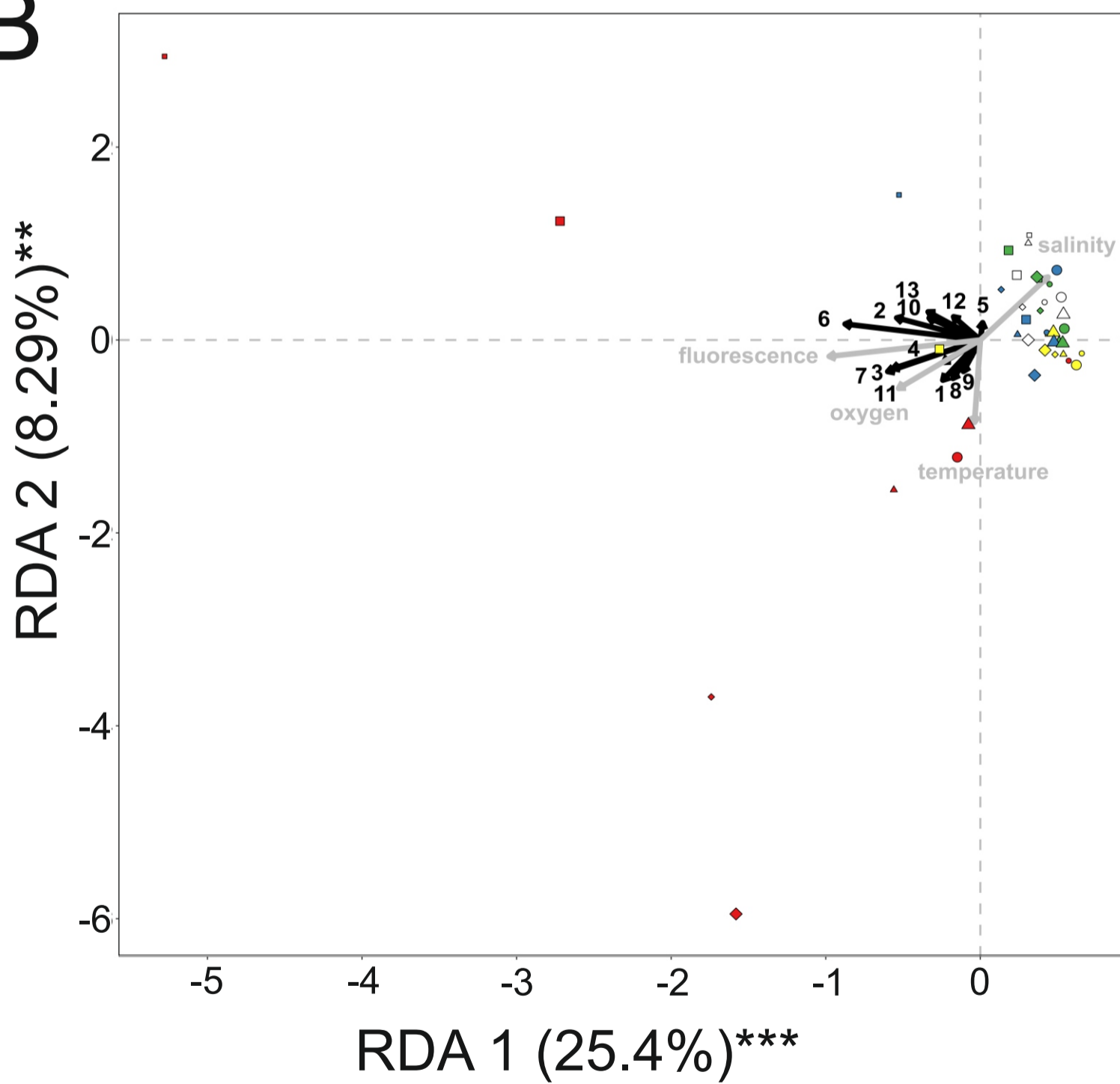

Supplement: Supplemental Information 1 — RDA ordination for (A) abundance and (B) biovolume of the most important zooplankton groups constrained by four variables. In the RDA correlation biplot, angles among explanatory (grey arrows) and/or response (black arrows) variables reflect their correlations. Depth strata are represented by color of data points, day/night sampling (size of data points) and seasons (symbols). Taxa code: 1 Amphipoda, 2 Calanidae, 3 Calanoida (<1.5 mm), 4 Chaetognatha, 5 Cnidaria, 6 Copepoda (nauplii), 7 Cyclopoida, 8 Euchaetidae, 9 Euphausiacea, 10 Harpacticoida, 11 Metridinidae, 12 Mysida, 13 Ostracoda. [file peerj-10-12823-s001.pdf]
